# The Intraoperative Difficulty Spectrum of Laparoscopic Cholecystectomy: A Stepwise Analysis of Operative Challenges and Safe Cholecystectomy Strategies

**DOI:** 10.7759/cureus.114513

**Published:** 2026-08-13

**Authors:** Jaspreet Shergill, Harmandeep S Jabbal, Priyanka Sharma, Jyoti Sharma, Nikhil Garg, Aryan Bikonia

**Affiliations:** 1 General Surgery, All India Institute of Medical Sciences, Bathinda, IND; 2 Pathology, All India Institute of Medical Sciences, Bathinda, IND; 3 Anaesthesiology, All India Institute of Medical Sciences, Bathinda, IND; 4 Surgical Oncology, All India Institute of Medical Sciences, Bathinda, IND

**Keywords:** bailout procedures, completion cholecystectomy, critical view of safety, difficult cholecystectomy, laparoscopic cholecystectomy, near-infrared fluorescence cholangiography, safe cholecystectomy

## Abstract

Background and objective

Laparoscopic cholecystectomy (LC) has become the preferred surgical approach for the excision of a diseased gallbladder because of its minimally invasive nature, enhanced safety profile, reduced postoperative complications, faster recovery, and shorter hospital stays. The Society of American Gastrointestinal and Endoscopic Surgeons (SAGES) Safe Cholecystectomy Program focuses on preventing bile duct injury (BDI) by emphasizing the importance of standardized dissection methods, the Critical View of Safety (CVS), intraoperative imaging, and bailout procedures to reduce complications during LC. Difficult gallbladders remain a significant technical challenge and are associated with an increased risk of BDI. This study aimed to evaluate the stepwise intraoperative challenges encountered during LC and assess the role of contemporary safe cholecystectomy principles in the management of difficult, complex, and reoperative biliary surgery. It also aimed to identify the challenges encountered during LC and improve outcomes through the application of current safe cholecystectomy practices.

Materials and methods

This study was conducted over one year, from April 2024 to March 2025, at the Department of General Surgery, All India Institute of Medical Sciences (AIIMS), Bathinda, Punjab, India. Difficult LC was defined as an operative duration of more than 60 minutes, inability to safely achieve the CVS due to severe inflammation, and/or the need for advanced dissection or laparoscopic bailout procedures. Intraoperative challenges were analysed according to six operative steps: creation of capnoperitoneum, adhesiolysis, achievement of the CVS, cystic pedicle control, dissection of the gallbladder from the cystic plate, and specimen retrieval. The use of near-infrared fluorescence cholangiography (NIRF-C), laparoscopic bailout procedures, and outcomes in complex and reoperative biliary cases were also evaluated.

Results

Of the 442 patients, 156 (35.3%) underwent difficult LC. The most common intraoperative challenge was adhesiolysis (74.4%), followed by difficulty in achieving the CVS (55.1%), specimen retrieval (30.8%), creation of capnoperitoneum (23.1%), and dissection of the gallbladder from the cystic plate (23.1%). Male sex, acute cholecystitis, previous abdominal surgery, abnormal hepatocystic anatomy, intrahepatic gallbladder, and large or multiple gallstones were the principal factors associated with operative difficulty and prolonged operative time (106.39 ± 21.66 minutes). Laparoscopic bailout procedures were successfully employed when indicated. No BDI, major vascular injury, bowel injury, mortality, or conversion to open surgery was observed. Complex biliary pathologies, including gangrenous or perforated gallbladder, cholecystoenteric fistula, and selected completion cholecystectomies, were successfully managed using a laparoscopic approach.

Conclusions

A structured, stepwise approach to LC, combined with routine demonstration of the CVS, selective use of NIRF-C, and timely adoption of laparoscopic bailout procedures, facilitates the safe management of difficult and complex biliary disease while minimizing major complications and preserving the benefits of minimally invasive surgery. The proposed stepwise intraoperative safety algorithm provides a practical framework to guide decision-making during difficult LC.

## Introduction

Gallstone disease remains a major global health concern, contributing to substantial morbidity and mortality. Gallstones develop within the gallbladder or bile ducts as a result of metabolic disturbances. In the United States, more than 20 million individuals are affected, contributing to a substantial number of hospital admissions each year [1]. In India, the prevalence is approximately 6% of the population, with a higher prevalence among women than men (10% vs. 3%) [2].

Laparoscopic cholecystectomy (LC) is a minimally invasive and widely preferred surgical technique for the treatment of symptomatic gallstones [3]. However, despite its advantages, LC may be associated with various intraoperative challenges, including extensive adhesions, gallbladder wall thickening, anatomical variations such as abnormal cystic arteries or ducts, and large gallstones, which may necessitate conversion to open surgery [4]. Minor complications, such as hemorrhage and bile spillage, can generally be managed effectively, whereas major complications, including bile duct injury (BDI) and major vascular injuries, can be life-threatening. BDI remains one of the most feared complications of LC and is associated with significant morbidity and mortality, medicolegal implications, and impaired quality of life. The incidence of complications following LC ranges from 0.5% to 6%, with BDI (0.1-0.6%) and major vascular injuries (0.04-1.22%) representing some of the most serious complications. Gallbladder perforation with spillage of bile and gallstones is another common intraoperative complication, occurring in 10-30% of patients [5].

Contemporary safe cholecystectomy practices have evolved substantially following the publication of the Society of American Gastrointestinal and Endoscopic Surgeons (SAGES) Safe Cholecystectomy Program and multisociety consensus guidelines [6-8]. The modern approach emphasizes the routine identification of the Critical View of Safety (CVS), avoidance of excessive traction and misidentification errors, recognition of hazardous anatomy and adherence to “stopping rules,” liberal use of intraoperative cholangiography (IOC) or near-infrared fluorescence cholangiography (NIRF-C), early adoption of bailout procedures when the CVS cannot be safely achieved, and a structured culture of intraoperative pause and effective team communication.

With increasing surgical experience and wider adoption of laparoscopic techniques, surgeons are increasingly confronted with complex biliary pathology including gangrenous or perforated gallbladder, empyema, cholecystoenteric fistula, and reoperative biliary surgery following abandoned or subtotal cholecystectomy. These conditions are characterized by severe inflammation, dense adhesions, distorted hepatocystic anatomy, and loss of normal tissue planes, making laparoscopic dissection technically demanding and increasing the risk of bile duct and vascular injury. Nevertheless, advances in laparoscopic instrumentation, high-definition imaging, NIRF-C [9], advanced energy devices, and the adoption of standardized safe cholecystectomy principles have enabled successful laparoscopic management of many of these complex cases in experienced centers [7-13]. Careful patient selection, meticulous surgical technique, and timely adoption of bailout procedures remain essential for achieving optimal outcomes while preserving the benefits of minimally invasive surgery [14].

Hence, we conducted this retrospective observational study to systematically evaluate the stepwise intraoperative challenges encountered during LC, identify factors associated with difficult dissection, and describe the operative strategies used to safely manage these challenges. In addition, we present an intraoperative decision-making framework that integrates our institutional experience with contemporary safe cholecystectomy principles and current consensus recommendations. This framework is intended as a practical guide and requires prospective multicenter validation before wider clinical adoption.

## Materials and methods

All adult patients undergoing an attempted LC over a period of one year from April 2024 to March 2025 at the Department of General Surgery, All India Institute of Medical Sciences (AIIMS), Bathinda, Punjab, India, were prospectively maintained in a departmental database and retrospectively analyzed. The study included 442 patients who presented to the hospital and met our inclusion criteria.

Inclusion criteria

The inclusion criteria were as follows: adult patients (≥ 18 years) undergoing LC during the study period; patients undergoing elective or emergency LC for symptomatic cholelithiasis, acute or chronic calculous cholecystitis, gallbladder empyema, mucocele of the gallbladder, and gallstone pancreatitis after resolution of the acute episode; patients with difficult gallbladder pathology requiring advanced laparoscopic dissection, including contracted or scleroatrophic gallbladder, intrahepatic gallbladder, Mirizzi syndrome, and cholecystoenteric fistula; patients undergoing completion LC for symptomatic remnant gallbladder or stump cholelithiasis following a previous abandoned or subtotal cholecystectomy performed elsewhere; and patients in whom LC was attempted, irrespective of whether bailout procedures (subtotal cholecystectomy or conversion to open surgery) became necessary.

Exclusion criteria

The exclusion criteria were as follows: patients younger than 18 years; patients undergoing planned open cholecystectomy without an initial laparoscopic attempt; patients with suspected or histologically proven gallbladder carcinoma; patients undergoing cholecystectomy as part of another major hepatobiliary or pancreatic procedure; and patients unfit for general anesthesia or with contraindications to laparoscopy in whom LC was not attempted.

Operative technique

All LCs were performed using a standardized four-port technique by surgeons with more than 10 years of experience in minimally invasive surgery. Capnoperitoneum (CP) was established using a Veress needle (12-15 mmHg), followed by placement of the remaining ports under direct vision with the patient in reverse Trendelenburg position and right-side elevation. A structured, stepwise approach to safe cholecystectomy was adopted in all cases. The procedure included creation of pneumoperitoneum, adhesiolysis, meticulous dissection of the hepatocystic triangle, demonstration of CVS, secure ligation and division of the cystic artery and cystic duct, dissection of the gallbladder from the cystic plate, and specimen retrieval using an endoscopic retrieval bag. A deliberate intraoperative pause was performed to confirm the CVS before clipping and dividing the cystic structures. NIRF-C using intravenous indocyanine green (ICG) was selectively employed in all patients classified as having difficult LC to facilitate real-time delineation of extrahepatic biliary anatomy during dissection.

Whenever the CVS could not be achieved safely because of severe inflammation or distorted anatomy, laparoscopic bailout procedures were employed in accordance with contemporary safe cholecystectomy principles. These included fundus-first cholecystectomy, subtotal fenestrating or reconstituting cholecystectomy, cystic duct stump suture ligation, or tube cholecystostomy, depending on the intraoperative findings. Careful haemostasis, irrigation, and retrieval of the specimen were performed to complete the procedure. Operative time was recorded from creation of CP to completion of skin closure. Difficult LC was defined as an operative duration exceeding 60 minutes and/or the requirement for advanced dissection or laparoscopic bailout procedures.

Intraoperative challenges were prospectively assessed at six predefined operative steps: creation of CP, adhesiolysis, achievement of CVS, cystic pedicle control, gallbladder dissection from the cystic plate, and specimen retrieval. The requirement for bailout procedures, conversion to open surgery, intraoperative complications, and overlapping technical difficulties were also recorded and included in the final analysis. When anatomical factors contributed to difficulty in obtaining CVS, each case was assigned to a single mutually exclusive category based on the predominant intraoperative finding. Abnormal hepatocystic anatomy included atypical anatomical relationships of the gallbladder and Calot's triangle other than isolated cystic duct or vascular anomalies. Short and wide cystic duct and anomalous vessel were classified as independent categories. In patients with multiple concurrent anatomical variations, classification was based on the factor considered by the operating surgeon to be the principal cause of difficulty.

The proposed intraoperative decision-making framework was not used to guide patient management during the study period. Rather, it was developed after completion of the retrospective analysis by integrating the observed intraoperative findings with contemporary safe cholecystectomy principles and published consensus recommendations.

Statistical analysis

Data were entered into Microsoft Excel and analyzed using SPSS Statistics version 29.0 (IBM Corp., Armonk, NY). Continuous variables were expressed as mean ± standard deviation (SD), while categorical variables were presented as frequencies and percentages. Continuous variables, including age and operative duration, were compared between the easy and difficult LC groups using the independent-samples Student's t-test after confirming approximate normality of distribution. Categorical variables, including sex, BMI (> 30 kg/m²), acute cholecystitis, previous abdominal surgery, thick gallbladder wall, intrahepatic gallbladder, abnormal hepatocystic anatomy, large or multiple gallstones, and the requirement for bailout procedures, were compared using the Pearson chi-square test. Fisher's exact test was used whenever the expected cell frequency was less than five. The association between categorical variables and difficult LC was assessed using the Pearson chi-square test. Crude odds ratios (ORs) with 95% confidence intervals (CIs) were calculated to quantify the strength of the unadjusted association. A two-tailed p-value < 0.05 was considered statistically significant.

## Results

This study included 442 cases that underwent LC, of which 286 (64.71%) were completed within 60 minutes, while 156 (35.29%) were classified as difficult, requiring more than 60 minutes for completion. Male sex was significantly associated with difficult LC. Of the 144 male patients, 72 (50.0%) underwent difficult LC, compared with 84 of 298 female patients (28.2%). Male patients had significantly higher unadjusted odds of difficult LC than female patients (crude OR: 2.55, 95% CI: 1.68-3.88; p < 0.001) (Table 1).

**Table 1:** Association between patient sex and difficult laparoscopic cholecystectomy (n = 442) Easy LC was defined as an operative duration of ≤ 60 minutes, whereas difficult LC was defined as an operative duration > 60 minutes and/or the need for advanced dissection or laparoscopic bailout procedures. Association between sex and difficult laparoscopic cholecystectomy was assessed using the Pearson chi-square test. Crude ORs with 95% CIs were calculated using female sex as the reference category LC: laparoscopic cholecystectomy; OR: odds ratio; CI: confidence interval

Sex	Easy LC, n (%)	Difficult LC, n (%)	OR (95% CI)	Test statistics, p-value
Female	214 (71.8)	84 (28.2)	1.00 (reference)	—
Male	72 (50.0)	72 (50.0)	2.55 (1.68–3.88)	χ²(1, N = 442) = 20.23, < 0.001

**Figure 1:**
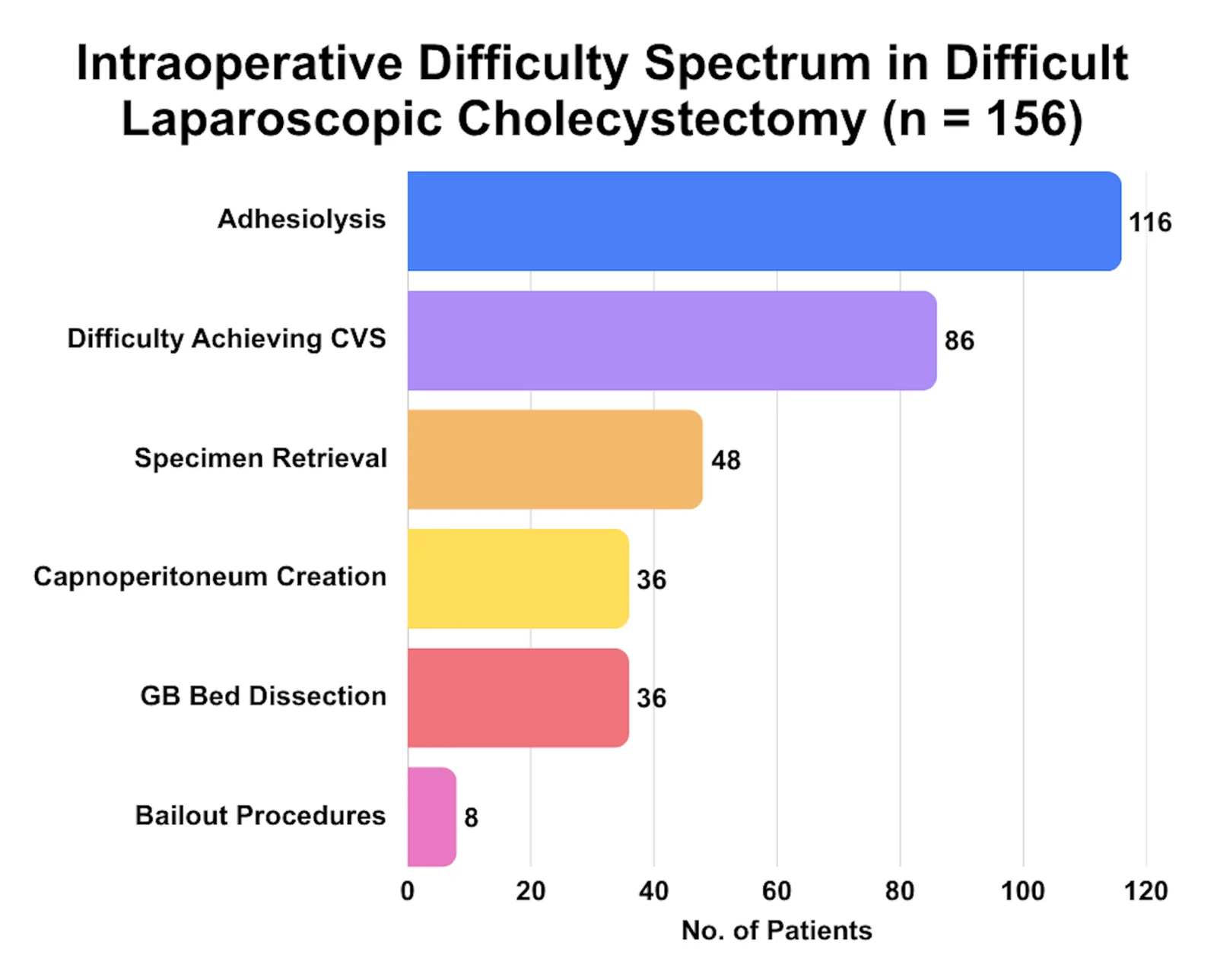
Intraoperative difficulty spectrum in difficult laparoscopic cholecystectomy (n = 156) Patients may have more than one difficulty during surgery. Percentages were calculated using the total cohort (n = 156) as the denominator CVS: Critical View of Safety; GB: gallbladder

In terms of the intraoperative difficulty spectrum in difficult LC (Figure 1), CP formation was difficult in 36 (23.1%) of the 156 challenging cases. The majority of patients with prior abdominal surgery experienced difficulty during CP creation (Table 2). Adhesiolysis was the most common difficulty, encountered in 116 patients (74.4%) of the 156 difficult cases. Patients with acute cholecystitis had the highest incidence of adhesions (46; 39.7%). Demonstration of the CVS and ligation and division of the cystic artery and duct were challenging in 86 (55.1%) of the 156 difficult cases (Table 2).

**Table 2:** Intraoperative difficulty spectrum and bailout procedures used in difficult laparoscopic cholecystectomy (n = 156) Percentages are calculated within each operative step. Only descriptive statistics are presented, and no inferential statistical comparisons were performed CVS: Critical View of Safety; ERCP: endoscopic retrograde cholangiopancreatography

Operative step	Cause of difficulty/bailout procedure	N	%
Capnoperitoneum creation (n = 36)	Previous abdominal surgery	18	50.0
	Morbid obesity	16	44.4
	Peritonitis	2	5.6
Adhesiolysis (n = 116)	Acute cholecystitis	46	39.7
	Post-ERCP cholangitis	26	22.4
	Previous abdominal surgery	20	17.2
	Empyema gallbladder	14	12.1
	Acute pancreatitis	10	8.6
Difficulty achieving CVS (n = 86)	Abnormal hepatocystic anatomy	44	51.2
	Short and wide cystic duct	34	39.5
	Anomalous vessel	8	9.3
Gallbladder bed dissection (n = 36)	Intrahepatic gallbladder	20	55.6
	Small contracted gallbladder	16	44.4
Specimen retrieval (n = 48)	Large/multiple calculi	18	37.5
	Thick-walled/rigid gallbladder	16	33.3
	Large distended gallbladder	14	29.2
Bailout procedures (n = 8)	Fundus-first approach	4	50.0
	Subtotal cholecystectomy	3	37.5
	Tube cholecystostomy	1	12.5
	Conversion to open surgery	0	0

**Figure 2:**
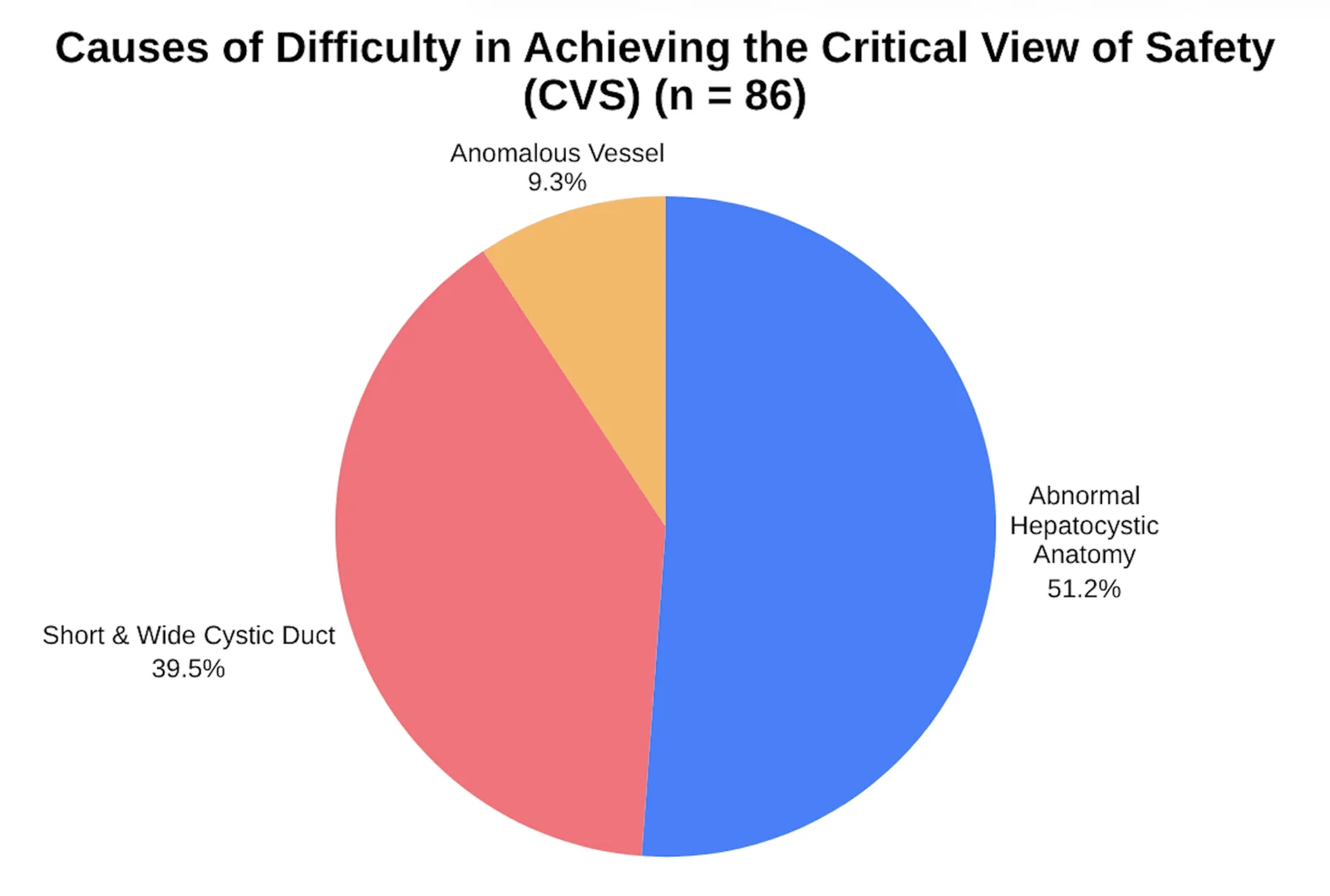
Causes of difficulty in achieving the CVS (n = 86) Primary anatomical factors responsible for difficulty in achieving CVS. Each patient was assigned to a single, mutually exclusive category based on the predominant intraoperative factor contributing to the difficulty CVS: Critical View of Safety

**Figure 3:**
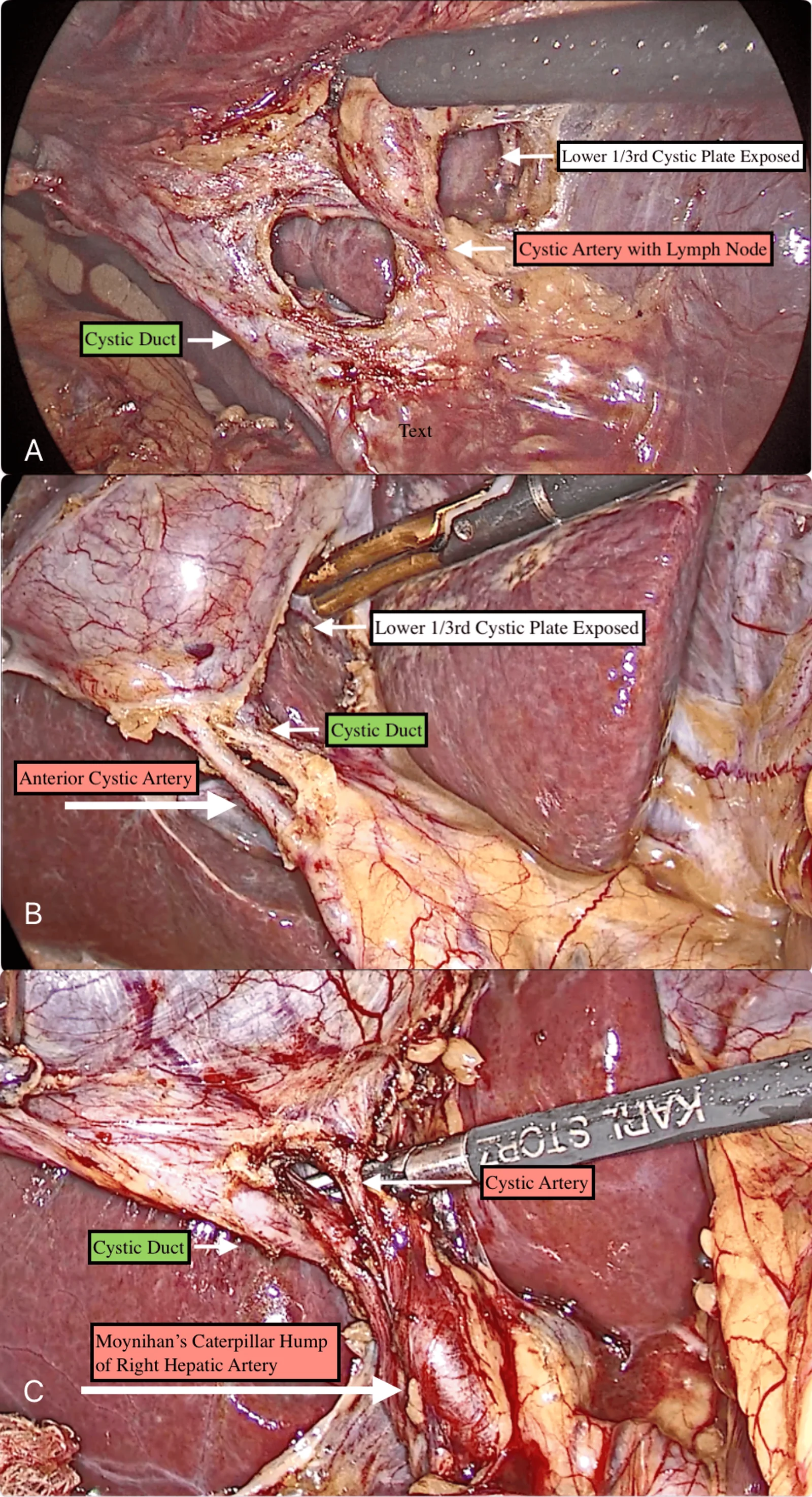
Critical View of Safety in varied anatomy (A) Acute cholecystitis showing an enlarged lymph node of Lund: inflammation poses a significant difficulty during demonstration of the CVS. (B) Anterior cystic artery: this variation occurs when the cystic artery is encountered anterior to the cystic duct, rather than in its normal position in the hepatocystic triangle. (C) Moynihan’s caterpillar hump of the right hepatic artery with the short cystic artery: this variation is important to recognize early to avoid injury and its devastating complications CVS: Critical View of Safety

**Figure 4:**
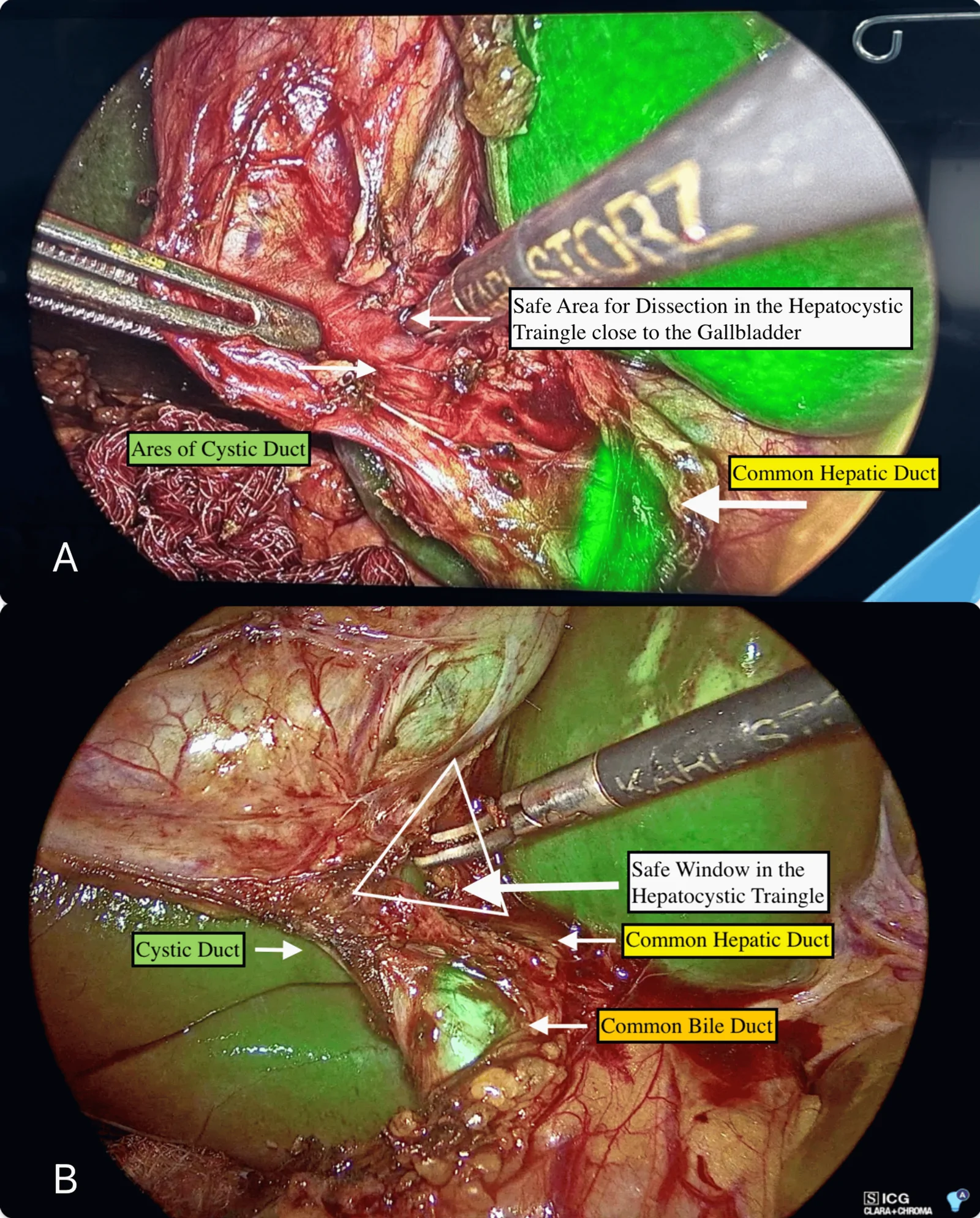
Intraoperative NIRF-C with ICG Representative intraoperative image demonstrating the anatomical relationships during laparoscopic cholecystectomy. The CHD, CD, and other relevant structures are directly labelled. The recommended safe area of dissection is outlined with boundary markings and anatomical landmarks to facilitate interpretation and illustrate the principles of safe cholecystectomy. (A) CHD is highlighted with NIRF-C for safe dissection. (B) Safe area of dissection in the hepatocystic triangle for demonstration of CVS under NIRF-C guidance NIRF-C: near-infrared fluorescence cholangiography; ICG: indocyanine green; CHD: common hepatic duct; CD: cystic duct; CVS: Critical View of Safety

Patients with abnormal hepatocystic anatomy, 44 (51.2%), showed the greatest difficulty during this step of LC (Figure 2). A standardized technique, as described above, was used with meticulous dissection to demonstrate the CVS (Figure 3). Advanced laparoscopic bipolar energy devices and NIRF-C with ICG (Figure 4) facilitated the management of difficult cases. NIRF-C was successfully utilized in all 156 difficult laparoscopic cholecystectomy cases (100%) as part of our institutional protocol for managing difficult biliary dissection. Because all difficult cases received NIRF-C, an internal comparison was not possible to assess its independent effect on operative outcomes or complication rates. In 36 (23.1%) patients, dissection of the gallbladder from the liver bed was challenging, mainly due to an intrahepatic gallbladder in 20 (55.6%) patients. Gallbladder extraction through the anterior abdominal wall was difficult in 48 patients (30.8%), due to large or multiple gallstones in 18 (37.5%), a thickened wall in 16 (33.3%), and a large, distended gallbladder in 14 (29.2%) patients (Table 2).

Of the 442 patients included in the study, 286 (64.7%) underwent easy LC, while 156 (35.3%) underwent difficult LC (Table 3).

**Table 3:** Comparison between easy and difficult laparoscopic cholecystectomy Continuous variables were compared using the independent-samples Student's t-test. Categorical variables were compared using the Pearson chi-square test or Fisher's exact test where appropriate. OR with 95% CI was calculated for the association between male sex and difficult laparoscopic cholecystectomy using binary logistic regression. A two-tailed p-value < 0.05 was considered statistically significant, and p < 0.001 was highly significant. Additional analysis: male sex was significantly associated with difficult LC (OR: 2.55, 95% CI: 1.68–3.88; χ² = 20.23; p < 0.001; calculated using binary logistic regression). Easy LC is defined as operative duration ≤ 60 minutes without advanced dissection or bailout procedures. Difficult LC is defined as operative duration > 60 minutes and/or requirement for advanced dissection or laparoscopic bailout procedures LC: laparoscopic cholecystectomy; SD: standard deviation; BMI: body mass index; GB: gallbladder; OR: odds ratio; CI: confidence interval

Variable	Easy LC (n = 286)	Difficult LC (n = 156)	P-value
Age, years, mean ± SD	42.5 ± 3.27	51.2 ± 3.12	< 0.001
Male sex, n (%)	72 (25.2)	72 (46.2)	< 0.001
Female sex, n (%)	214 (74.8)	84 (53.8)	
BMI ≥ 25 kg/m², n (%)	94 (32.9)	68 (43.6)	< 0.05
Acute cholecystitis, n (%)	34 (11.9)	61 (39.1)	< 0.001
Previous abdominal surgery, n (%)	14 (4.9)	28 (17.9)	< 0.001
Thick GB wall (> 4 mm), n (%)	40 (14.0)	64 (41.0)	< 0.001
Multiple/large stones, n (%)	51 (17.8)	58 (37.2)	< 0.001
Intrahepatic gallbladder, n (%)	3 (1.05)	20 (12.8)	< 0.001
Abnormal hepatocystic anatomy, n (%)	9 (3.15)	44 (28.2)	< 0.001
Completion LC for abandoned/residual gallbladder	0	18 (11.5)	—

The mean operative duration among difficult LC cases was 106.39 ± 21.66 minutes. The most frequent factors associated with prolonged operative duration were acute cholecystitis (39.1%), abnormal hepatocystic anatomy (28.2%), previous abdominal surgery (17.9%), intrahepatic gallbladder (12.8%), and large or multiple calculi (11.5%). Multiple factors often coexisted in the same patient. Patients who underwent difficult LC were generally older than those in the easy LC group, with a mean age of 51.2 ± 3.12 years compared with 42.5 ± 3.27 years (p < 0.05). Difficult procedures were also encountered more frequently in male patients. Males constituted 46.2% of the difficult LC group compared with 25.2% of the easy LC group, and male sex was associated with a 2.55-fold higher likelihood of a difficult procedure (OR: 2.55, 95% CI: 1.68-3.88; p < 0.001).

Several preoperative factors were found to be significantly associated with operative difficulty. Obesity (BMI ≥ 25 kg/m²) was more common among patients with difficult LC (43.6% vs. 32.9%; p < 0.05). Similarly, a history of previous abdominal surgery was significantly more frequent in the difficult group (17.9% vs. 4.9%; p < 0.001). Inflammatory changes involving the gallbladder were strongly associated with increased operative difficulty. Acute cholecystitis was present in 39.1% of patients with difficult LC, compared with only 11.9% of those with easy LC (p < 0.001). Likewise, a thickened gallbladder wall (> 4 mm) was observed nearly three times as frequently in difficult cases (41.0% vs. 14.0%; p < 0.001).

**Figure 5:**
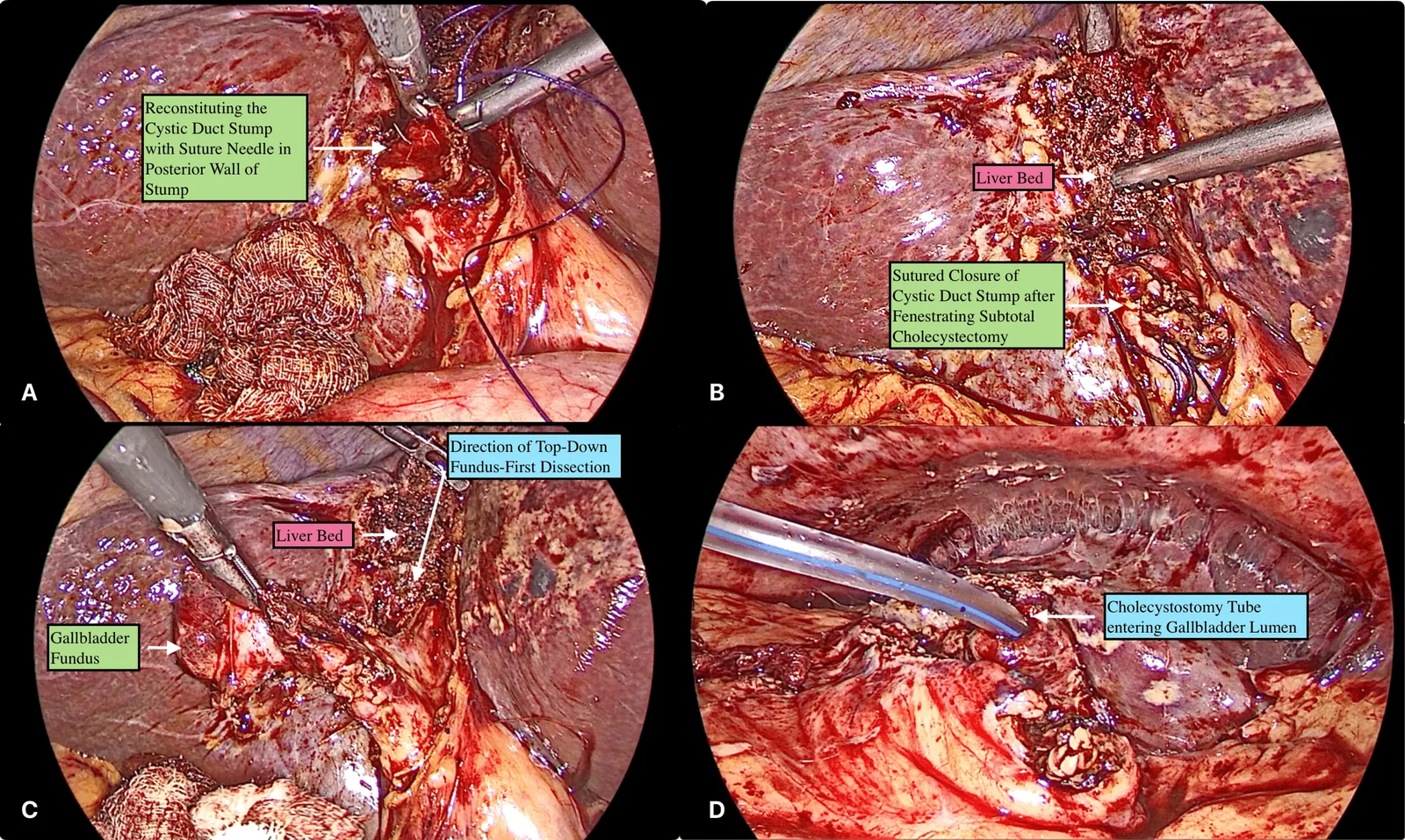
Laparoscopic bailout procedures Representative intraoperative images illustrating commonly employed laparoscopic bailout strategies in difficult cholecystectomy. (A) Reconstituting subtotal cholecystectomy demonstrating the remnant gallbladder and closure of the gallbladder stump. (B) Fenestrating subtotal cholecystectomy with closure of the cystic duct/gallbladder remnant, highlighting the residual gallbladder wall and dissection plane. (C) Fundus-first (top-down) cholecystectomy illustrating the direction of dissection from the gallbladder fundus toward the infundibulum; fundus-first (top-down) cholecystectomy performed as a bailout strategy after failure of the conventional antegrade approach. (D) Tube cholecystostomy demonstrating placement of the drainage tube within the gallbladder remnant. Arrows and superimposed labels identify the principal anatomical structures and the key operative steps in each bailout technique

The calculus burden also appeared to influence surgical complexity. Multiple or large gallstones were identified in 37.2% of difficult LC cases, compared with 17.8% of easy cases (p < 0.001). Anatomical variations showed some of the strongest associations with operative difficulty. An intrahepatic gallbladder was present in 12.8% of difficult procedures, compared with only 1.05% of easy procedures (p < 0.001). Similarly, abnormal hepatocystic anatomy was encountered in over one-quarter of difficult cases (28.2%), compared with just 3.15% of easy cases (p < 0.001). Notably, all eight bailout procedures performed in the difficult LC group were successful (Figure 5). One patient who underwent bailout with tube cholecystostomy subsequently underwent interval LC at eight weeks and had an uneventful recovery.

Eighteen out of 156 patients (11.5%) in the difficult LC group were referred after a previously abandoned LC or subtotal cholecystectomy with stump cholelithiasis performed elsewhere. All patients underwent an uneventful completion LC at our center. These reoperative procedures constituted a particularly challenging subset owing to dense postoperative adhesions and altered hepatocystic anatomy.

Overall, difficult LC was significantly associated with older age, male sex, obesity, previous abdominal surgery, acute cholecystitis, a thickened gallbladder wall, larger or multiple gallstones, adverse anatomical factors including an intrahepatic gallbladder and abnormal hepatocystic anatomy, as well as reoperative procedures such as completion cholecystectomy. These findings indicate that inflammatory changes, anatomical variations, and reoperative surgery are major contributors to operative difficulty (Table 3). Sixty-eight (43.6%) of the 156 patients who underwent difficult LC presented with advanced biliary pathology that significantly increased the technical complexity of LC. These included gallbladder empyema, a contracted scleroatrophic gallbladder associated with cirrhosis, gallbladder perforation, a gangrenous gallbladder, cholecystoduodenal fistula, and reoperative completion LC (Table 4).

**Table 4:** Complex gallbladder pathology encountered among difficult laparoscopic cholecystectomies (n = 156) Percentages are calculated using the difficult laparoscopic cholecystectomy group (n = 156) as the denominator. Patients may have more than one complex biliary pathology; therefore, the pathological categories are not mutually exclusive, and the frequencies should not be summed to represent the total number of patients LC: laparoscopic cholecystectomy

Pathology	N	% of difficult LC
Empyema gallbladder	12	7.7
Contracted scleroatrophic gallbladder	11	7.1
Gallbladder perforation	14	9
Gangrenous gallbladder	8	5.1
Cholecystoenteric fistula	5	3.2
Stump cholelithiasis/previous abandoned cholecystectomy	18	11.5

**Figure 6:**
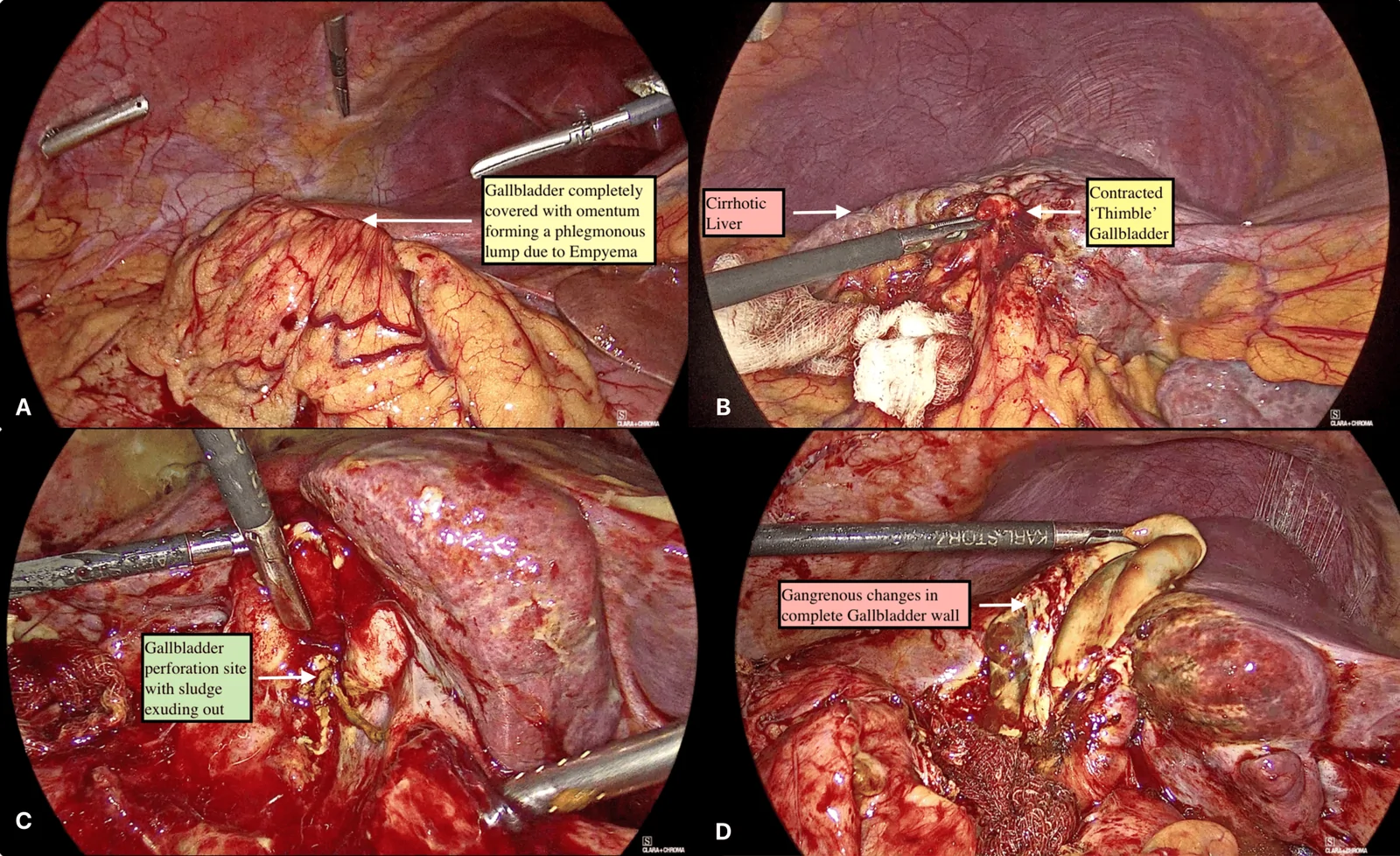
Complex gallbladder pathology encountered during laparoscopic cholecystectomy (A) Empyema of the gallbladder showing a tense, distended gallbladder completely concealed by omentum with inflammatory changes. (B) Contracted, scleroatrophic gallbladder in a cirrhotic liver demonstrating dense fibrosis and obliteration of tissue planes. (C) Gallbladder perforation with the perforation site (arrow) and surrounding inflammatory changes. (D) Gangrenous gallbladder demonstrating areas of necrotic gallbladder wall (arrow) with associated inflammatory changes. Arrows and superimposed labels identify the principal pathological features in each panel

Empyema gallbladder (Figure 6A) and gangrenous gallbladder (Figure 6D) were characterized by dense inflammatory adhesions, oedematous tissues, and distorted hepatocystic anatomy, requiring meticulous adhesiolysis and careful dissection for safe completion of the procedure. Gallbladder perforation (Figure 6C) was associated with localized bile contamination and inflammatory adhesions, while contracted scleroatrophic gallbladders (Figure 6B) posed additional technical challenges due to dense fibrosis, difficulty with grasping, and challenges in achieving the CVS.

**Figure 7:**
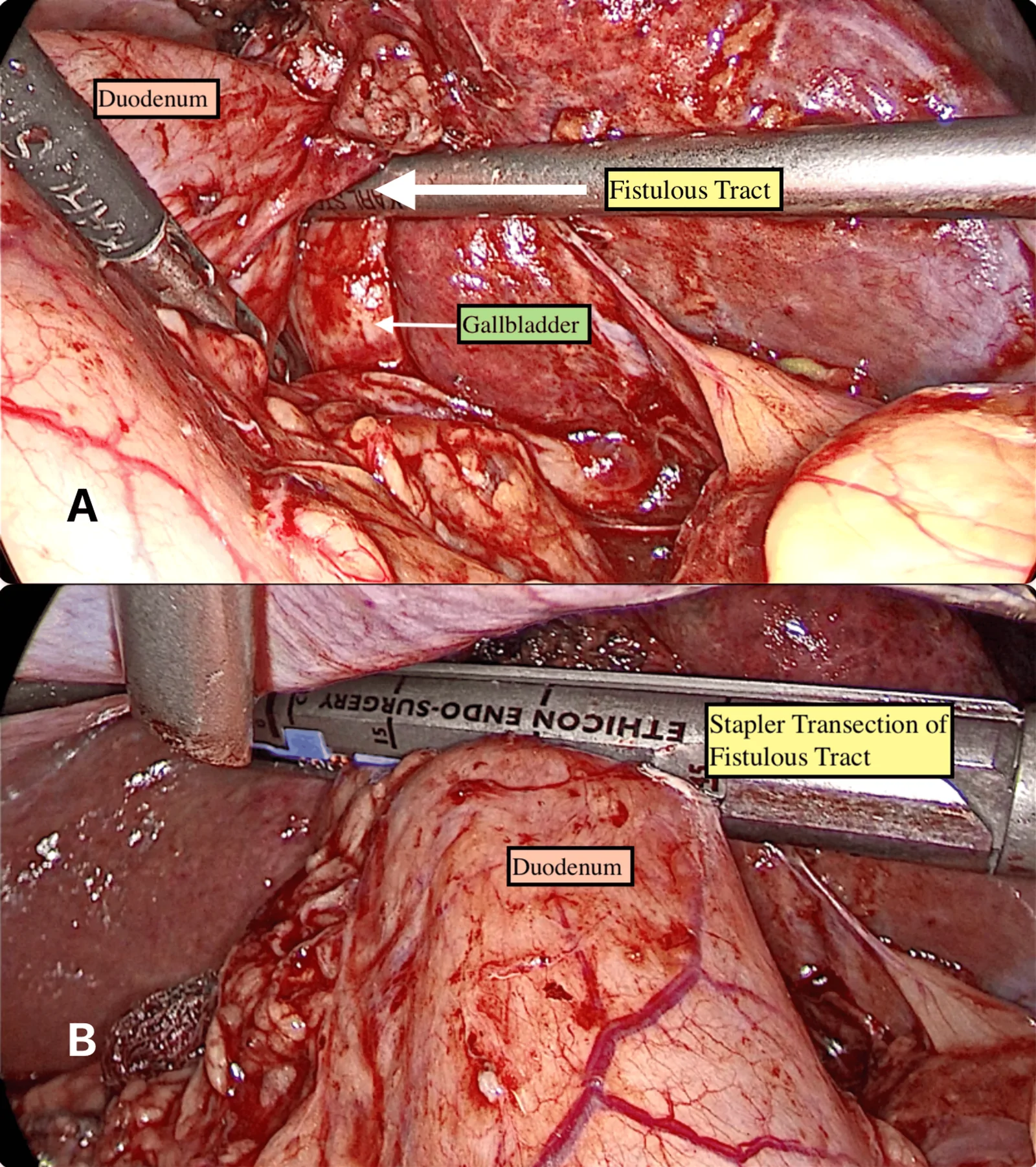
Cholecystoenteric fistula managed laparoscopically (A) Dissection and isolation of the cholecystoduodenal fistula demonstrating the gallbladder remnant, duodenum, and fistulous tract before division. (B) Stapled resection of the cholecystoduodenal fistula. Arrows and superimposed labels identify the principal anatomical structures and operative steps

Five patients with cholecystoenteric fistula were successfully managed laparoscopically. Following meticulous adhesiolysis, the fistulous tract was carefully isolated (Figure 7A) and divided using an endoscopic linear stapler (Figure 7B). Four patients had cholecystoduodenal fistulae, while one had a cholecystocolic fistula.

**Figure 8:**
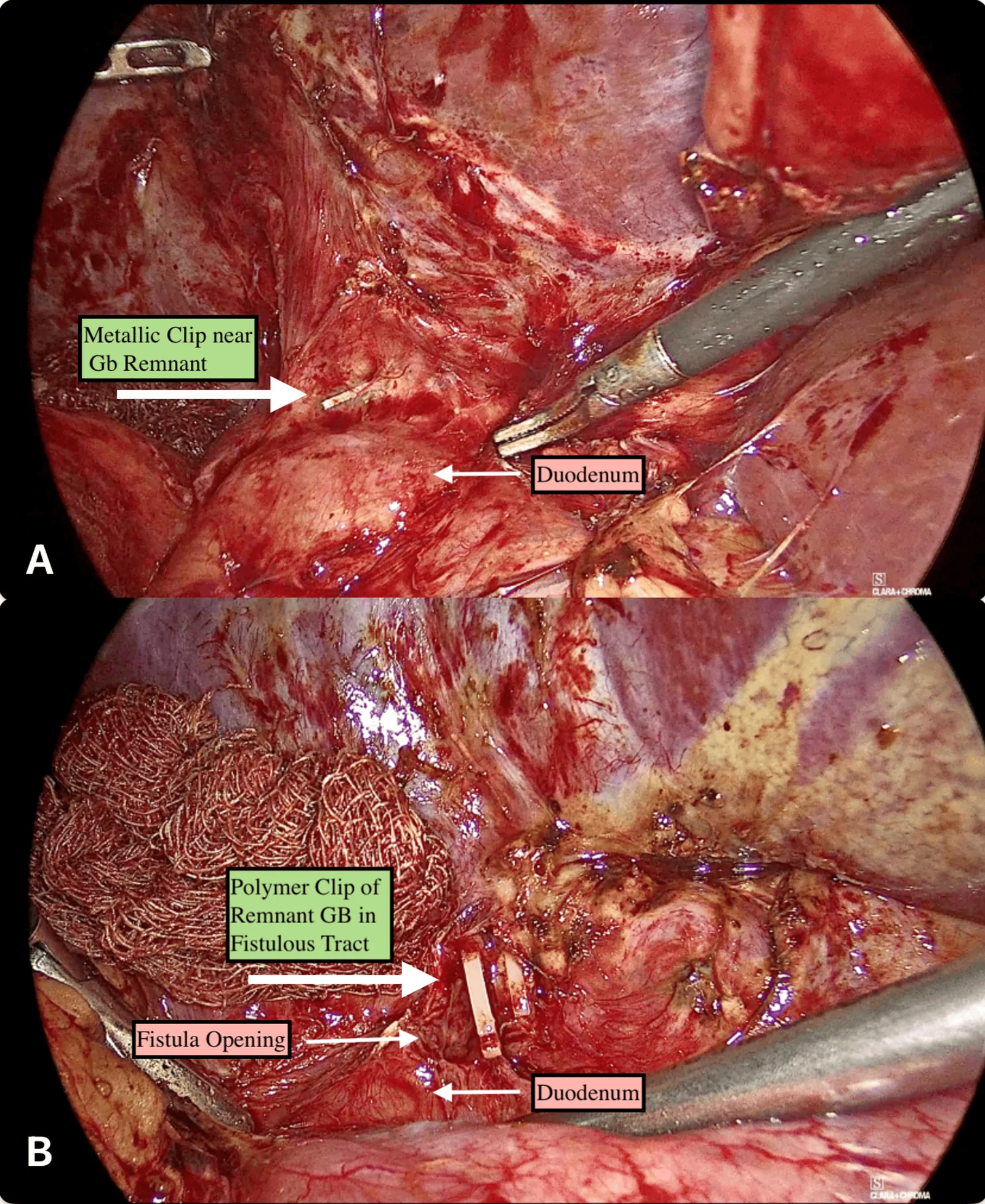
Completion laparoscopic cholecystectomy for residual gallbladder disease (A) Previous metallic clips over the cystic pedicle identified during reoperative dissection. (B) Residual gallbladder with a cholecystoduodenal fistula and a previously placed polymer clip. Arrows and superimposed labels identify the remnant gallbladder, fistulous communication, duodenum, and previous polymer clip

**Figure 9:**
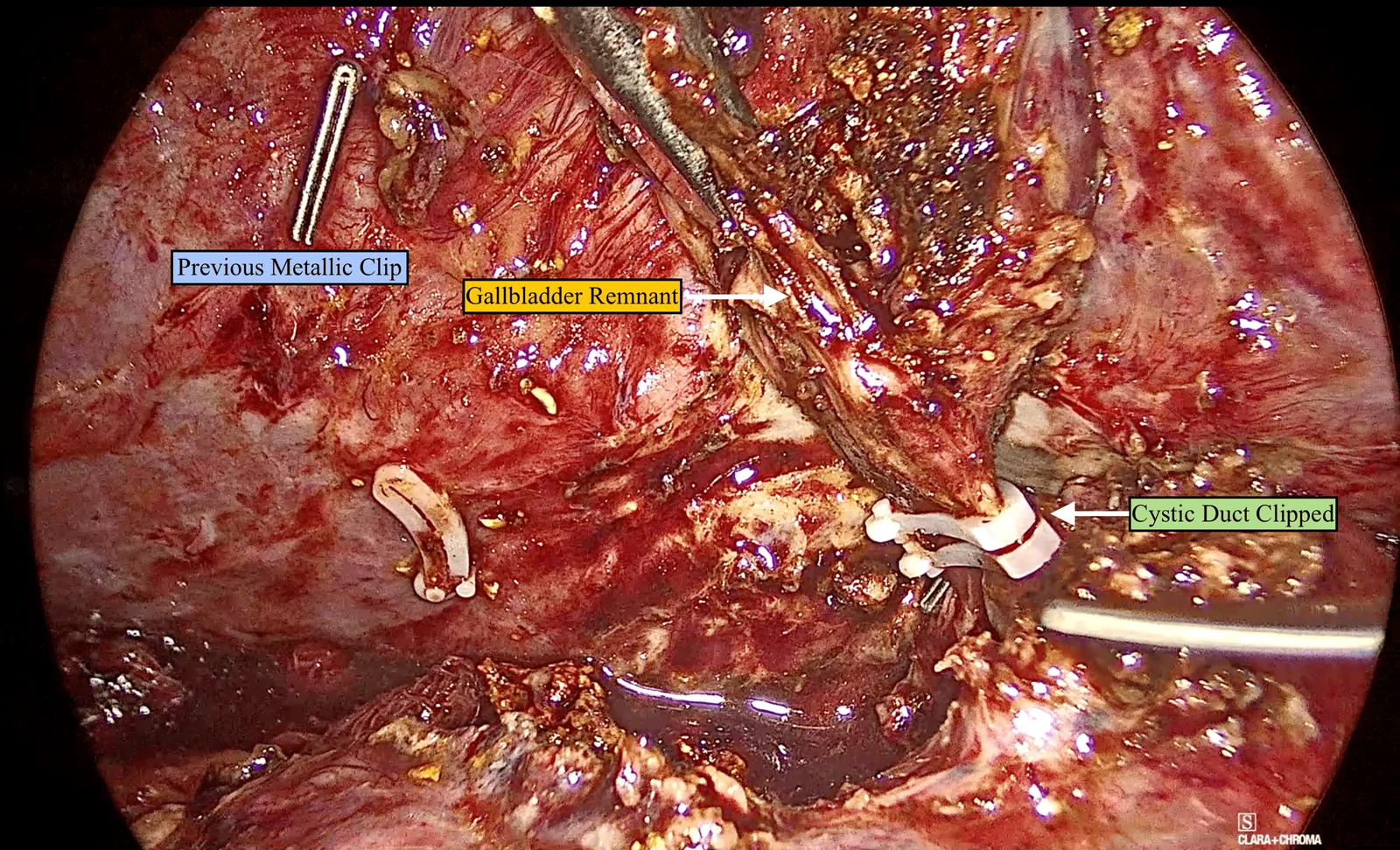
Completion laparoscopic cholecystectomy Intraoperative image demonstrating the CVS achieved during completion laparoscopic cholecystectomy for stump cholelithiasis following previous subtotal cholecystectomy. The cystic duct is shown after complete circumferential dissection and ligation. Arrows and superimposed labels identify the CD, remnant gallbladder, previous surgical clips, new ligation clips, and the structures defining the CVS CVS: Critical View of Safety; CD: cystic duct

Eighteen patients underwent completion LC for symptomatic residual gallbladder calculi following previous subtotal or abandoned cholecystectomy. Dense adhesions, fibrosis, altered hepatocystic anatomy, and previously placed metallic and polymer clips were encountered during reoperative dissection (Figure 8). Following meticulous adhesiolysis, the CVS was achieved, allowing secure ligation and division of the cystic duct (Figure 9). All procedures were completed laparoscopically without major intraoperative complications or conversion to open surgery.

Although the primary focus of this study was the characterization of intraoperative challenges and the strategies used to address them, major intraoperative adverse events were also documented. No bile duct injuries, major vascular injuries, or bowel injuries occurred, and no patient required conversion to open surgery. In selected difficult cases, laparoscopic bailout strategies facilitated safe completion of the procedure. No major postoperative complications, including bile leak requiring intervention, major hemorrhage, reoperation, or postoperative mortality, were observed during the study period. One patient who underwent tube cholecystostomy during the index procedure subsequently underwent successful interval LC eight weeks later.

## Discussion

This study was conducted to assess the outcomes of LC in a total of 442 cases, comprising 286 easy and 156 difficult cases. Challenges encountered in difficult cases were the formation of CP (23.07%) in patients with prior abdominal surgeries, extensive adhesion separation (74.36%) in cases of acute cholecystitis, manipulation of arteries and ducts (55.13%) in patients with abnormal hepatocystic anatomy, excision of the gallbladder (23.08%) in cases of intrahepatic gallbladder, and extraction of the gallbladder (30.77%) in cases of gallbladders with a high concentration of stones. These factors were also associated with a longer operative duration (106.39 ± 21.66 minutes) in difficult cases. These findings provide valuable insights into the challenges encountered during LC and highlight the importance of careful patient selection and precise surgical technique for achieving optimal outcomes.

Establishing CP, which involves accessing the abdominal cavity followed by CO₂ insufflation to provide an optimal working platform and visualization, is the first stage of LC. We routinely establish CP using the Veress needle technique. In patients with scarring from previous abdominal surgery, CP is established at Palmer’s point. Adequate CP can be established with a pressure of 12-15 mmHg [15]. Of the 442 patients in our study, 36 experienced difficulty during CP creation. Previous abdominal surgery (50%) and obesity (43.5%) were the major contributing factors. Creating CP with a Veress needle and inserting laparoscopic trocars in patients with previous upper abdominal surgery may place the visceral organs at risk of injury due to adhesions between the parietes. Palmer’s point is a safe alternative in such cases, with trocars inserted under direct visualization. An open approach using the Hasson technique may also be employed for CP creation in these patients [16].

The increasing complexity of accessing the gallbladder, along with ongoing inflammation and extensive adhesion formation, can result in prolonged operative duration. In our study, the most significant difficulty during LC was observed in patients with abnormal hepatocystic anatomy, which was present in 44 cases (51.16%). Kiviluoto et al. [17] conducted a randomized trial comparing LC and open cholecystectomy for acute and gangrenous cholecystitis. They reported that 16% of patients in the laparoscopic group required conversion to open surgery because severe inflammation resulted in distortion of the anatomy of the HC triangle. In another study by Lal et al. [18], which included 73 patients, 11 patients (15.07%) with contracted gallbladders encountered difficulties during LC, and five of these patients required conversion to open surgery.

However, in our study, no patient required conversion to open surgery, as all patients were successfully managed using laparoscopic techniques. This finding should be interpreted with caution and should not be taken to suggest that conversion to open surgery can always be avoided. Conversion to open surgery is an accepted and appropriate bailout strategy when safe laparoscopic dissection cannot be achieved and should never be regarded as a surgical failure. The absence of conversion in our cohort most likely reflects the routine use of contemporary safe cholecystectomy principles, including fundus-first dissection, subtotal cholecystectomy, tube cholecystostomy, and other laparoscopic bailout techniques, performed by experienced surgeons at a high-volume tertiary referral center. Therefore, our findings should not be interpreted as evidence of technical superiority, but rather as reflecting the structured application of safe cholecystectomy strategies within this specific institutional setting.

In the present study, bailout procedures were required in only eight (5.1%) patients despite the inclusion of 156 difficult LCs. Fundus-first (top-down) cholecystectomy was the most frequently employed bailout strategy (4/8, 50.0%), followed by subtotal cholecystectomy (3/8, 37.5%) and tube cholecystostomy (1/8, 12.5%), while no patient required conversion to open surgery. Following tube cholecystostomy during the index operation, one patient underwent planned interval LC eight weeks later. Although fundus-first dissection is fundamentally an alternative direction of gallbladder dissection, in our practice it was not used routinely but was selectively adopted only when conventional antegrade dissection was deemed unsafe because of severe inflammation, dense adhesions, or distorted hepatocystic anatomy that precluded safe achievement of the CVS. Accordingly, we classified fundus-first dissection as a bailout strategy, as it represented a deliberate intraoperative change in surgical strategy intended to safely complete the procedure while minimizing the risk of BDI. The selective use of a stepwise bailout approach, together with adherence to safe cholecystectomy principles, likely contributed to the absence of conversion to open surgery in our series.

One important vascular variation encountered during LC is Moynihan's (caterpillar) hump of the right hepatic artery (Figure 3C). In this anomaly, the right hepatic artery follows a tortuous course within the hepatocystic triangle, resulting in a short cystic artery and close proximity of the artery to the cystic duct. This anatomical variation increases the risk of inadvertent vascular injury or misidentification during dissection, particularly in the presence of inflammation or distorted anatomy. Awareness of such vascular anomalies further emphasizes the importance of a structured, stepwise approach to safe LC. Recognition of this variant, meticulous dissection close to the gallbladder wall, routine demonstration of CVS, selective use of adjuncts such as NIRF-C, and timely employment of laparoscopic bailout procedures formed the cornerstone of our institutional strategy for managing difficult LC. In the present study, NIRF-C was used in all 156 difficult cases as part of our institutional protocol. However, as there was no comparison group managed without NIRF-C, our study was not designed to evaluate its independent contribution to operative outcomes. 

Patients with small, contracted scleroatrophic gallbladders or trabeculated gallbladders (Figure 6B), resulting from a heavy calculus burden and chronic cholecystitis, are particularly challenging and may make it difficult for the surgeon to securely grasp and manipulate the gallbladder [19]. In our study, we identified 16 cases (10.25%) with small, contracted gallbladders. The gallbladder may be fully or partially encased within the liver parenchyma congenitally, or may become buried within the liver as a result of recurrent inflammation. The absence of an avascular plane of dissection between the gallbladder and liver parenchyma, together with the inability to grasp the gallbladder fundus, can make dissection technically challenging [20]. We encountered 20 cases (12.82%) with intrahepatic gallbladders, all of which presented with difficulty during dissection from the cystic plate. Empyema gallbladder (Figure 6A) is a severe form of acute cholecystitis associated with suppuration. Malik et al. [21] reported that the difficulty of performing LC may not be as great as reported in other studies, particularly when performed by experienced surgeons. They reported that 80.59% of patients with gallbladder empyema underwent successful LC. We observed similar results, as all patients with empyema of the gallbladder in our study underwent successful LC.

Gallbladder perforation (Figure 6C) represents an advanced stage of acute cholecystitis and is frequently associated with severe inflammation, dense pericholecystic adhesions, localized abscess formation, and distortion of the hepatocystic triangle. These factors substantially increase the technical complexity of LC and the risk of BDI. Although gallbladder perforation has traditionally been considered an indication for conversion to open surgery, increasing surgical expertise together with adherence to modern safe cholecystectomy principles has enabled successful laparoscopic management in carefully selected patients [7,10]. Early control of bile contamination, meticulous adhesiolysis, evacuation of localized collections, and careful identification of anatomical landmarks are essential. When inflammation precludes safe demonstration of CVS, bailout procedures such as subtotal cholecystectomy or tube cholecystostomy should be adopted without hesitation to prevent major biliovascular injury [7,14].

Gangrenous gallbladder (Figure 6D) is another formidable challenge during LC because of tissue friability, transmural necrosis, edema, and obliteration of normal tissue planes. The friable gallbladder wall frequently tears during grasping, resulting in bile spillage and loss of effective traction, while extensive inflammation obscures the cystic duct-artery junction. Previous studies have shown that gangrenous cholecystitis is associated with prolonged operative time, higher conversion rates, and increased postoperative morbidity [11,13]. Nevertheless, experienced laparoscopic surgeons can safely complete most procedures by employing gentle traction, advanced bipolar energy devices for meticulous haemostasis, liberal use of NIRF-C with indocyanine green or intraoperative cholangiography whenever indicated, and timely adoption of bailout procedures when CVS cannot be confidently achieved [7,9,11].

Cholecystoenteric fistula (Figure 7), although uncommon, represents one of the most technically demanding situations encountered during LC. Chronic inflammation leads to dense adhesions between the gallbladder and adjacent bowel, most commonly the duodenum, hepatic flexure of the colon, or stomach. Safe laparoscopic management requires meticulous adhesiolysis with careful separation of the fistulous tract while avoiding enteric injury. Depending upon the degree of inflammation, laparoscopic fistula division with intracorporeal repair of the bowel defect can be successfully done in experienced hands; however, conversion to an open procedure remains entirely appropriate whenever safe laparoscopic dissection is not feasible [10,11]. Contemporary safe cholecystectomy guidelines emphasize that conversion should never be regarded as a complication but rather as sound surgical judgment undertaken to prevent catastrophic biliovascular or enteric injury [7].

The successful management of these advanced inflammatory conditions further reinforces the importance of a structured stepwise approach to LC. Routine pursuit of CVS, liberal use of NIRF-C with ICG where available, judicious utilization of advanced energy devices, and early employment of laparoscopic bailout strategies collectively enhance operative safety while minimizing unnecessary conversion and reducing the incidence of major BDI [6,7,9]. Completion LC following a previously abandoned cholecystectomy, subtotal cholecystectomy, or residual gallbladder stump with calculi (Figures 8, 9) is another technically demanding procedure that surgeons may increasingly encounter. Patients may present with recurrent biliary pain, acute cholecystitis, choledocholithiasis, pancreatitis, or persistent post-cholecystectomy symptoms due to retained gallbladder tissue or cystic duct stump calculi [7,12]. Dense adhesions, fibrosis, and distorted anatomy around the hepatocystic triangle make these reoperative procedures particularly challenging and increase the risk of BDI.

Traditionally, completion cholecystectomy was performed through an open approach because of concerns regarding difficult dissection and altered anatomy. However, with increasing laparoscopic expertise and advances in imaging and energy devices, completion LC has become a safe and feasible option in experienced hands [12,13]. Careful preoperative evaluation with magnetic resonance cholangiopancreatography (MRCP) and selective endoscopic retrograde cholangiopancreatography (ERCP), along with intraoperative adjuncts such as NIRF-C using indocyanine green, can help delineate biliary anatomy and facilitate safe dissection [9,12].

The principles of safe cholecystectomy remain equally important in these reoperative cases. Meticulous adhesiolysis, dissection close to the gallbladder remnant, routine pursuit of CVS, and early use of bailout procedures whenever safe anatomy cannot be established are essential to minimize the risk of major biliary or vascular injury [6-8]. Several studies have demonstrated that completion LC provides excellent symptomatic relief with low morbidity and should be considered the preferred approach whenever expertise is available [12,13]. Finally, gallbladder extraction can be difficult in cases with a thickened gallbladder and large stones. In such instances, extending the fascial incision may be necessary to facilitate the gallbladder's removal [16]. In our study, out of 156 difficult cases, 48 (30.8%) had difficulty in extracting the gallbladder from the abdominal wall. The greatest difficulty during this step of LC was seen in gallbladders having a high density of calculi (18, 37.5%). 

Overall, our findings underscore that difficult LC is a dynamic process in which operative complexity progressively evolves throughout the different stages of the procedure. Our experience corroborates existing literature, demonstrating that even the most challenging LCs can be safely completed using a structured, stepwise approach. Recognition of adverse pathology, meticulous dissection, routine demonstration of the CVS, appropriate use of adjuncts such as NIRF-C, and timely employment of laparoscopic bailout procedures are the cornerstones of safe surgery. Equally important is the willingness to modify the operative strategy according to intraoperative findings rather than persisting with unsafe dissection. Conversion to open surgery should be regarded as a valid safety strategy when laparoscopic progress cannot be achieved safely. The absence of conversion in our series likely reflects surgeon experience, careful patient selection, and liberal use of laparoscopic bailout procedures, rather than suggesting that conversion is unnecessary. These principles not only facilitate the successful completion of difficult and reoperative cholecystectomies but also collectively help minimize major biliary and vascular complications and optimize patient outcomes [22-24].

**Figure 10:**
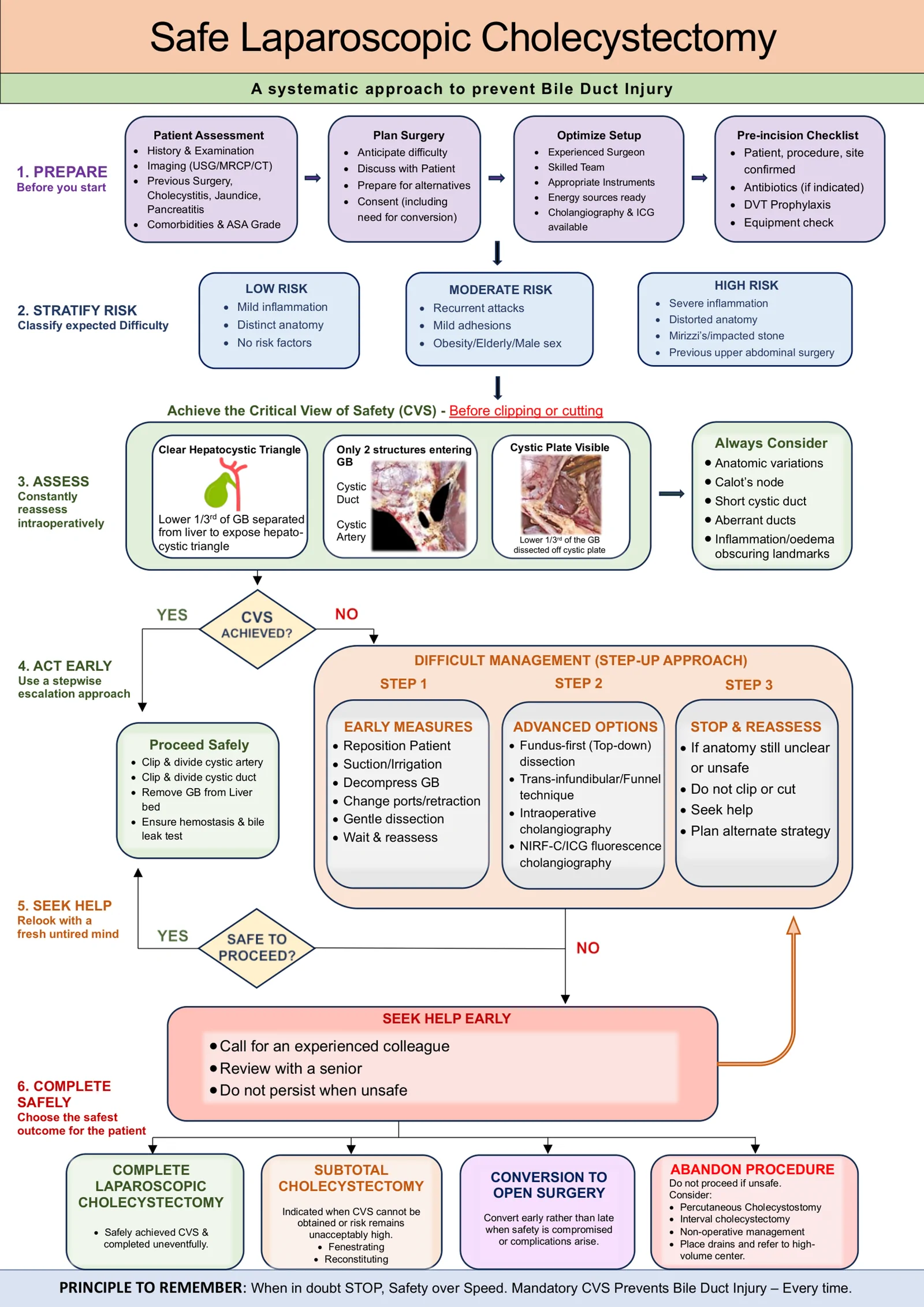
Proposed institutional stepwise intraoperative workflow for safe laparoscopic cholecystectomy Stepwise intraoperative algorithm for safe laparoscopic cholecystectomy incorporating CVS, time-out checkpoints, escalation strategies, NIRF-C/ICG fluorescence cholangiography, and bailout procedures to minimize bile duct injury. Developed by the authors based on current consensus practices, including the principles of the CVS, SAGES Safe Cholecystectomy Multi-Society Practice Guideline, Tokyo Guidelines 2018, the Universal Culture of Safety in Cholecystectomy, and contemporary evidence regarding NIRF-C/ICG fluorescence cholangiography and laparoscopic bailout strategies [6-9,11,14,22-24] CVS: Critical View of Safety; NIRF-C: near-infrared fluorescence cholangiography; ICG: indocyanine green; SAGES: the Society of American Gastrointestinal and Endoscopic Surgeons Algorithm flowchart figure created in Microsoft Office Word 2021 by the corresponding author

Based on our institutional experience and contemporary safe cholecystectomy principles, we propose a stepwise intraoperative workflow (Figure 10) that integrates preoperative risk assessment, continuous intraoperative reassessment, structured pause checkpoints, early senior consultation, and timely transition to appropriate bailout strategies when safe anatomical identification cannot be achieved. This workflow is intended as an institutional decision-support framework rather than a universal standard of care. Although derived from a single-center retrospective experience, it may serve as a practical guide for intraoperative decision-making. Prospective multicentre studies involving larger and more diverse patient cohorts are required to validate its applicability, reproducibility, and clinical effectiveness before broader adoption can be recommended.

Limitations

The present study is limited by its single-center design and the absence of a comparative group. Furthermore, the procedures were performed by experienced laparoscopic surgeons in a high-volume tertiary care center, which may limit the generalizability of the findings to other settings. A standardized intraoperative grading system such as the Nassar scale was not incorporated because it was not part of the original retrospective study design. The authors relied on clinical judgement for defining operative difficulty, using a duration threshold (> 60 minutes) instead of using other validated clinical or anatomical severity grading systems. Thus, the chances of having operator-dependent bias cannot be ruled out. Although NIRF-C was routinely employed in all difficult LC cases, its independent effect on operative difficulty, conversion rate, or biliary complications could not be assessed because no control group without NIRF-C was available.

Future prospective comparative studies are required to determine its incremental clinical benefit. The reported ORs represent unadjusted associations. Multivariable logistic regression, which would allow adjustment for potential confounding variables such as age, acute cholecystitis, previous abdominal surgery, gallbladder inflammation, and anatomical variations, could not be performed because the original patient-level dataset was unavailable. Therefore, the findings should be interpreted as crude associations rather than independent predictors of difficult LC. Furthermore, the proposed workflow was developed from a single-center retrospective experience and has not been prospectively validated. Its performance, reproducibility, and clinical utility should be evaluated in larger multicenter cohorts before it can be recommended for widespread implementation. Future multicenter studies with multivariate analysis are needed to validate these observations using a validated clinical grading system.

## Conclusions

Difficult LC is most commonly encountered in patients of the male sex, acute cholecystitis, dense adhesions, previous abdominal surgery, abnormal hepatocystic anatomy, intrahepatic gallbladder, and large or multiple gallstones. Recognition of these factors and anticipation of technical challenges at each stage of the procedure facilitate appropriate modification of the operative strategy. Careful dissection with routine attainment of CVS, timely recognition of intraoperative difficulty, adherence to the principles of safe cholecystectomy, judicious use of appropriate intraoperative adjuncts, and laparoscopic bailout strategies facilitate safe completion of difficult LC. In our experience, many complex biliary conditions, including gangrenous or perforated gallbladder, cholecystoenteric fistula, and completion cholecystectomy, can often be managed laparoscopically using a structured, safety-first approach when appropriate expertise is available. However, patient safety should remain the overriding priority, and timely use of laparoscopic bailout procedures or conversion to open surgery, when indicated, represents sound surgical judgment rather than failure. Adherence to the principles of safe cholecystectomy and appropriate intraoperative decision-making are fundamental to minimizing biliary and vascular complications. Based on our institutional experience and current evidence, we propose an institutional stepwise intraoperative workflow to support surgical decision-making during difficult LC. This workflow is intended as a hypothesis-generating and educational framework rather than a universal standard of care. Prospective multicenter studies involving larger and more diverse patient populations are required to externally validate its reproducibility, safety, and clinical utility before it can be recommended for widespread clinical implementation.
